# Contemporary management of recurrent respiratory papillomatosis: a survey of Australian otolaryngologists

**DOI:** 10.1007/s00405-025-09944-2

**Published:** 2026-01-12

**Authors:** Anthony Rotman, Ayden Tchernegovski, Debra Phyland, Paul Paddle

**Affiliations:** 1 Department of Otolaryngology, Head and Neck Surgery, Monash Health, Melbourne, Australia; 2 Faculty of Medicine, Nursing, Health Sciences, Monash University, Melbourne, Australia

**Keywords:** Recurrent respiratory papillomatosis, Surgical Management, Vaccination, Australia, Fellowship training

## Abstract

**Purpose:**

To assess current experiences and practices in assessment and management of recurrent respiratory papillomatosis (RRP) in Australian Otolaryngologists, with a secondary aim to determine the impact of Laryngology fellowship training on these outcomes.

**Methods:**

An online survey was developed and emailed to practicing Australian Otolaryngologists, determined by membership and registration status with the Australian Society of Otolaryngology Head and Neck Surgeons (ASOHNS).

**Results:**

Seventy-four of 507 ASOHNS members completed the survey. Only 20.3% completed Laryngology fellowships (n=15/74). Those who saw <2 individual RRP patients/year were excluded, leaving 46 respondents. The majority (n=29/46) only operated on <5 patients per year, typically adults (n=37/46). Lasers overall were the most common instrument used in adult patients (n=17/37), whilst the microdebrider in those who managed juvenile disease (n=9/17). Adjuvant therapy was used in 74.4%, stroboscopy in 58.1% and voice therapy in 86.1%. Laryngology-trained respondents saw more patients, used stroboscopy, recommended vaccination, used laser and adjuvant therapy more than other respondents.

**Conclusion:**

Our findings reflect the current trends of pre-operative, intraoperative and post-operative management of RRP amongst Australian Otolaryngology consultants. Many of these practices are consistent with findings from international literature, except for in-office procedures. Despite being a relatively rare disease, RRP presents a significant disease burden to the global population emphasising the importance of how it is managed. In recent decades, contemporary management options such as new ablative and angiolytic lasers and adjuvant options such as intralesional bevacizumab and the HPV vaccination have changed the way we manage RRP, particularly amongst the laryngology fellowship trained subspecialty population.

## Introduction

Recurrent respiratory papillomatosis (RRP) is a benign condition characterised by multi-focal papillomatous growths on the mucosa of the respiratory tract [1]. A manifestation of human papilloma virus (HPV), the condition presents bi-modally in children and adulthood [2]. Juvenile onset RRP (JO-RRP) is most commonly diagnosed between ages 2–4 and is thought to be caused by vertical transmission of HPV at birth [3, 4]. The pathophysiology of adult-onset recurrent respiratory papillomatosis (AO-RRP) is not well understood, however its clinical course is classically less aggressive than JO-RRP [5]. Management goals in patients with RRP are centred around preserving voice quality and optimising airway patency, primarily through repeated surgical procedures with microdebrider, laser or cold steel instruments with complementary peri-operative voice therapy to optimise voice outcomes. Various adjuvant therapies are utilised to extend intervals between surgery and improve patient outcomes, including intraoperative intralesional injections and systemic treatments including vaccination.

Given the wide variety of treatment options and outcomes, and a disease process that is still poorly understood, this study aimed to survey current Australian Society of Otolaryngology Head and Neck Surgery (ASOHNS) consultants on their experience and practices in relation to RRP assessment and management. A secondary but important aim was to explore any differences in reported practices according to post-fellowship sub-specialty training and with a focus on Laryngology fellowship training, compared to non-Laryngology subspecialties. Finally, the Australian experience was compared to the current international literature.

## Methods

Approval for this study was granted by Monash University Human Research Ethics Committee (Project ID 31571). An online survey was developed with questions generated by the authors and using the platform Survey Monkey. The first seven questions sought to capture information related to participant demographics and included Australian region of practice, fellowship training, years of practice and experience managing RRP. The remaining questions were based around the workup, management and follow-up of RRP patients (List 1). Only participants who saw at least 2 patients per year with RRP were permitted to progress to this next part of the survey and subsequent questions were dependent on preceding responses (for example, the subset of vaccination questions were only asked of those who recommended the vaccine). The online survey link was sent to all currently practicing ASOHNS consultants via email. Consent was implicit if participants chose to complete the survey. The recruitment period was 6 weeks. Responses were anonymous and survey progression and completion was reliant on the nature of responses.

## Results

Results are described according to surgeon, patient and disease subcategories, as follows. Due to variations in the survey logic, sample size (n) varied as the survey progressed.

### Surgeon demographics

**Fig. 1:**
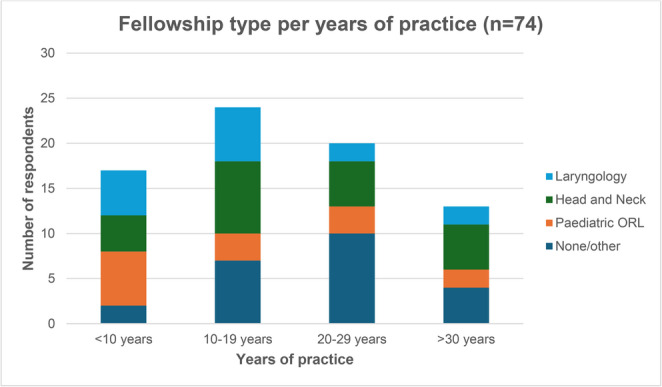
Fellowship type per years of practice

The response rate was 14.6% with 74 of the 507 ASOHNS members completing the survey. The majority of respondents were from New South Wales or Victoria (54%), had completed over 10 years of practice as a consultant (65%) and some form of fellowship training (82%) (see Table 1). Laryngology and head & neck surgery fellowships were the most common subspecialty training amongst those with < 20 years of consultant practice (*n* = 11/41 and *n* = 12/41, respectively) (Table 1; Fig. 1).

**Table 1:** Surgeon demographics

Demographic	*N* = 74 [Percentage of Respondents]
Region of Practice	
Australian Capital Territory	2 [2.7]
New South Wales	19 [25.7]
Northern Territory	0 [0]
Queensland	14 [18.9]
South Australia	6 [8.1]
Tasmania	2 [2.7]
Victoria	21 [28.4]
Western Australia	10 [13.5]
**Years since obtaining FRACS**	
< 10	17 [23.0]
10–19	24 [33.4]
20–29	20 [27.0]
>= 30	13 [17.6]
**Fellowship Training**	
No formal fellowship	13 [17.6]
Laryngology	15 [20.3]
Paediatric ORL	14 [18.9]
Head and Neck	22 [29.7]
Other	10 [13.5]

### Caseload characteristics

Over a third (37.8%) of all respondents saw < 2 individual RRP patients per year so were excluded from further analysis as they did not meet inclusion criteria.

### Assessment and management

Of the 46 respondents seeing 2 or more individual RRP patients per year, all operated on at least one patient per year and were therefore included in the remaining questions.

Almost all respondents more commonly operated on AO-RRP than JO-RRP patients (*n* = 42/45, 1 respondent failed to complete the survey at this juncture).

### Intraoperative care

#### Anaesthetic method and airway assessment

Microlaryngoscopy tubes (non-laser tube) and high-flow apnoeic nasal oxygen were the most preferred airway choices in those with low volume glottic disease (*n* = 13/45 and *n* = 14/45, respectively), while microlaryngoscopy tube, alone, was the most preferred airway choice in those with high volume glottic disease (*n* = 14/45).

Most respondents (90.9%) reported they performed intraoperative assessment of the trachea *often* or *always* while the main bronchi were assessed much less frequently with 64.4% (*n* = 29/45) of respondents assessing them *sometime*s or *not at all*. Intraoperative biopsy was performed variably with most clinicians only performing these *sometimes* (*n* = 29/45 [64.4%]). Of those performing biopsies, serotyping occurred *sometimes* (*n* = 19/43 [44.2%]) or *not at all* (*n* = 13/43 [30.2%]), with 2 respondents skipping this question for unknown reasons.

#### Surgical methods

**Fig. 2:**
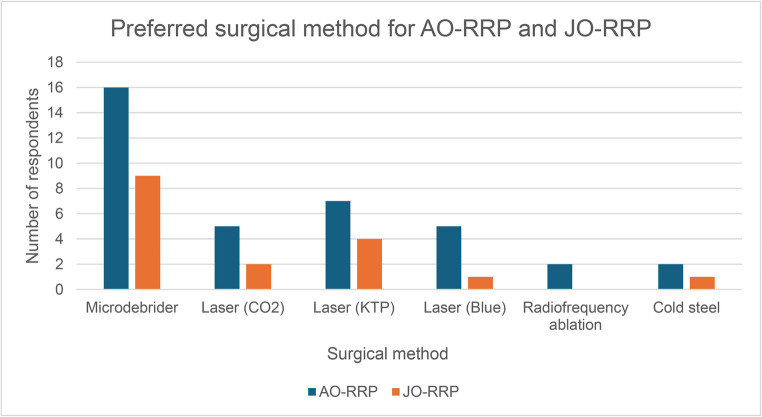
Preferred surgical method in JO-RRP and AO-RRP

Some respondents operated on AO-RRP or JO-RRP only, whilst others operated on both. As such, in 37 respondents who operate on AO-RRP, laser overall (including blue, CO_2_ or KTP) was reported to be the most commonly used method of surgery (*n* = 17/37) compared to microdebrider, radiofrequency ablation and cold steel, however microdebrider was the most popular individual method (*n* = 16/37). Of the 17 respondents who operate on JO-RRP, the microdebrider was the first choice as an individual tool (*n* = 9/17) (Fig. 2). Amongst those clinicians reporting they utilised laser as their first choice, KTP was preferred compared to CO2 and Blue light in both AO-RRP and JO-RRP groups. In patients with anterior commissure disease, 90.7% (*n* = 39/43) of respondents reported they would treat one side before completing a staged second procedure or re-assessing the patient.

#### Adjuvant therapies

The preference for intraoperative adjuvant therapies is notable, with 74.4% (*n* = 32/43) of respondents using them. Intralesional bevacizumab (62.5%) was preferred over intralesional cidofovir (31.3%). Of 21 respondents who used Bevacizumab, 71.5% used 25 mg/ml concentration and 28.5% used 2.5 mg/ml. Only 11/43 (25.6%) of respondents had referred patients for systemic Bevacizumab, mostly for those with aggressive tracheobronchial disease.

### Postoperative care

All but one clinician (97.7%) reported that they follow up patients that are macroscopically clear of disease after surgery. Over a third of respondents (*n* = 16/43) would vary their follow up intervals based on the extent of pre-operative disease with most clinicians seeing patients once to twice per year after surgery. Stroboscopy was used by 25/43 respondents in the assessment of glottic papilloma, the majority of these only sometimes (*n* = 10). Voice therapy is reported to be part of regular post-operative care for 37/43 clinicians.

### Vaccination

HPV vaccination was recommended by 93.1% (*n* = 27/29) and 81.0% (*n* = 34/42) of clinicians for patients with JO-RRP and AO-RRP, respectively. In juvenile onset patients, the 9-valent and quadrivalent vaccines were equally recommended (48.4% each) and the majority of practitioners waited until 12 years of age as per the National Immunisation Program protocol to make this recommendation (56.7%). In adults the quadrivalent was the recommended vaccine for 55.6% of respondents.

### Sub-group analysis based on fellowship

**Fig. 3:**
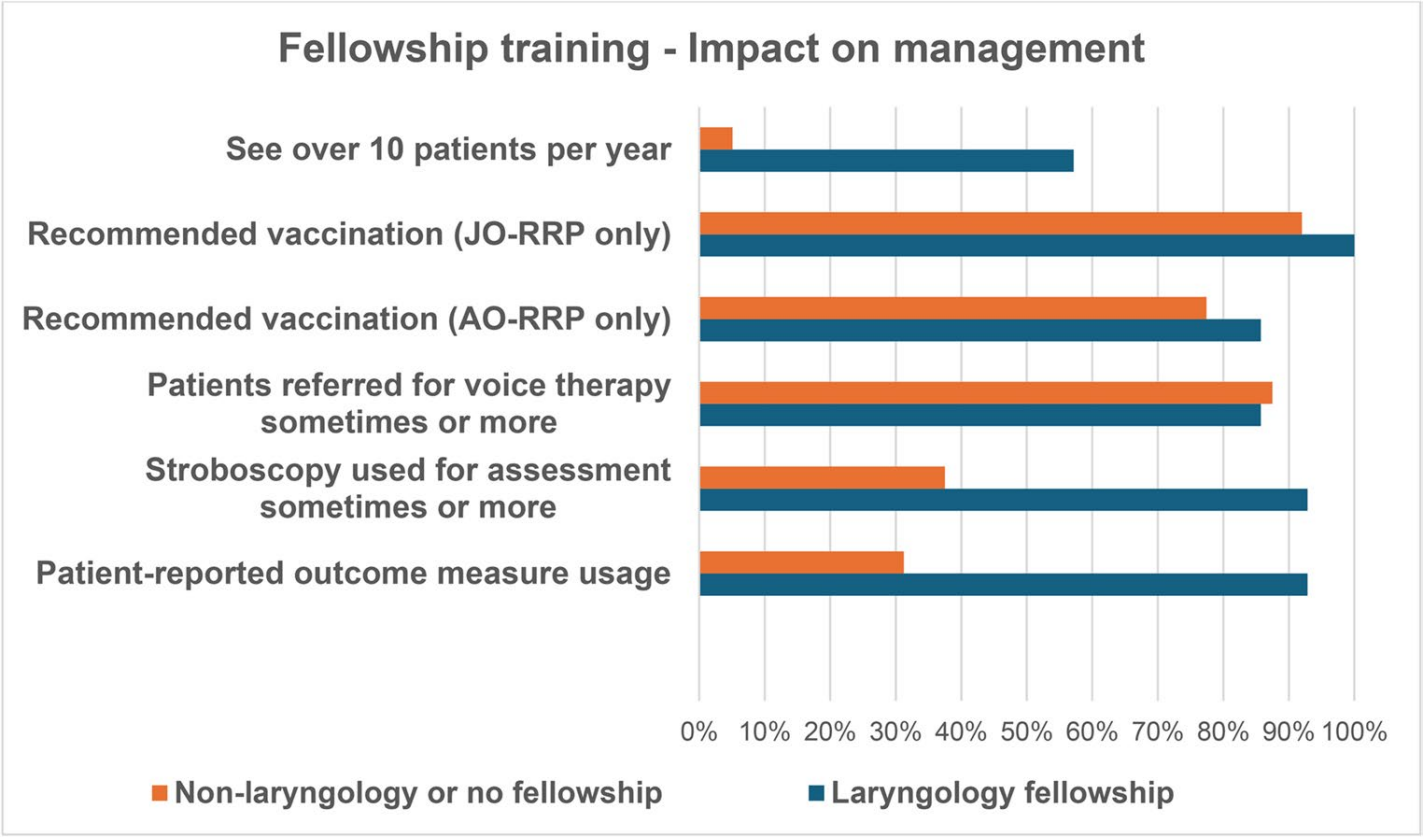
Subgroup analysis of management per fellowship training type, Laryngology or Non-laryngology/No fellowship

**Fig. 4:**
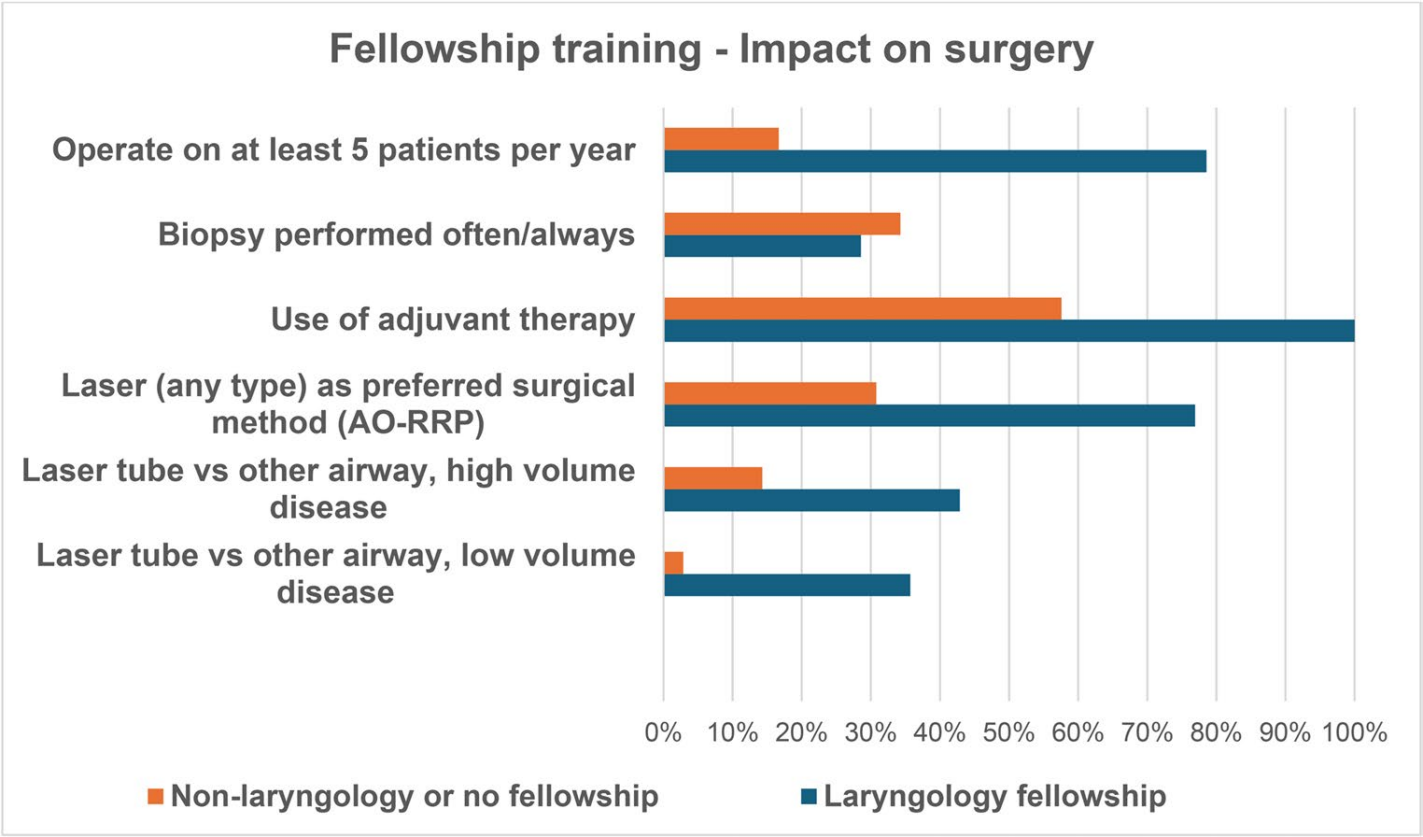
Subgroup analysis of impact on surgery per fellowship training type, Laryngology or Non-laryngology/No fellowship

A further analysis of results based on fellowship type was performed, with key questions assessed as per Figs. 3 and 4.

## Discussion

This study investigated the similarities and differences in the investigation, management and follow up of AO-RRP and JO-RRP amongst Otolaryngology consultants in Australia. While earlier studies have surveyed RRP management amongst otorhinolaryngologists in the UK [6], this study is the first to survey an Australian cohort. Moreover, a wider range of factors potentially impacting on clinical practice such as surgeon demographics, experience, training and caseload, as well as varied new treatment options were included in this survey compared to prior studies.

### Primary aim – current assessment and management of RRP by Australian surgeons

Historical surveys have demonstrated a low incidence of RRP overall with JO-RRP being twice as common as AO-RRP [7]. Importantly these surveys pre-date the widespread use of HPV vaccination with more recent surveys showing a significant decline in the prevalence of JO-RRP [8]. The relatively low overall prevalence of this disease was reflected in our survey responses. Almost 75% of all respondents saw less than 5 RRP individual patients per year and almost 65% operated on less than 5 RRP patients on an annual basis (Tables 2 and 3).

**Table 2:** Number of individual patients with RRP seen per year

RRP Patients Seen/Year	*N* = 74 [Percentage of Respondents]
< 2	28 [37.8]
2–4	27 [36.5]
5–9	8 [10.8]
10–19	8 [10.8]
> 20	3 [4.1]

**Table 3:** RRP patients operated on per year

RRP Patients Operated/Year	*N* = 46 [Percentage of Respondents]
< 5	29 [63]
5–9	9 [20]
10–19	7 [15]
> 20	1 [2]

One important omission from the survey relates to in-office procedures for RRP. Internationally, there is strong evidence supporting the safety and efficacy of office-based procedures for RRP [9], with some demonstrating improved cost effectiveness [10]. Specifically, a key challenge in the Australian context is the constrained level of training and access to in-office laryngology procedures. In addition, the availability of in-office lasers is limited by staffing requirements and safety regulations. As such, survey questions pertaining to surgical treatment relate to general anaesthetic only.

The choice of intraoperative airway is nuanced and will be governed by several factors including equipment availability, resection technique, anaesthetist preference, surgeon preference and volume of disease. Our survey established that microlaryngoscopy tubes were a popular surgeon choice in both low and high volume RRP. This is not surprising as microlaryngoscopy tubes have a smaller external diameter and grant better exposure of the surgical field compared to regular endotracheal tubes, based on clinician experience. Laser-safe microlaryngoscopy tubes may be harder to obtain, hence it is presumed non-laser microlaryngoscopy tubes would be more widely used despite the common use of laser as a surgical tool. It was hypothesised that, microlaryngoscopy tubes would be preferable for high volume disease as they provide wide surgical exposure, while still securing the airway and preventing distal spread of secretions and debris that occurs when resecting more extensive disease. More extensive disease also often extends operative time, hence a tube may be preferred to apnoeic techniques. Although tubeless options, such as high-flow nasal oxygen and jetting provide a completely uninterrupted view, they can be technically challenging and less effective in patients with reduced glottic patency like those with high volume RRP [11]. Another consideration is the theoretical risk of an airway fire when performing laser with high-flow oxygenation. It is expected that for these reasons, high-flow nasal oxygen was significantly more popular in low volume over high volume RRP. Laser safe endotracheal tubes were reported to be used more commonly as one may expect when laser was required. Laser exposure increases the risk of combustion through ignition of potential fuels including endotracheal tubes and nearby surgical materials, particularly in oxygen rich environments. Laser safe tubes aim to mitigate this risk through features such as fluid-filled double cuffs and laser resistant materials like silicone and foil wrapping [12]).

The importance of tracheal assessment in RRP patients has been highlighted by earlier studies demonstrating that a significant proportion of patients may have lower airways disease, in particular tracheal disease and in a small minority, pulmonary disease. Reported incidence rates amongst those with JO-RRP vary however previous studies show rates up to 23.3% for tracheal involvement and less than 5% for pulmonary involvement [13]. Reflecting this, most survey respondents reported they always performed intraoperative assessment of the trachea and uncommonly assessed the main bronchi.

Although respiratory papilloma has a characteristic macroscopic appearance on intraoperative airway assessment, the diagnosis of RRP occurs through biopsy with identification of distinguishing histopathological findings. As such the majority of respondents reported performing biopsy at least sometimes. Additional serotyping may be performed to identify HPV subtypes and risk-stratify patients. Repeat biopsies may also be considered in patients with risk factors for malignant transformation including high-risk HPV serotypes, dysplasia, aggressive recurrence, tracheobronchial disease, smoking and prior irradiation [14]. Overall, malignant transformation is uncommon and occurs in < 1% of all RRP patients however some risk factors such as pulmonary involvement leads to a much higher lifetime risk of up to 16% [15]. This study found that over two thirds of respondents who perform biopsies will obtain serotyping at least sometimes, likely in an attempt to identify the presence of high-risk HPV subtypes. It is not clear as to why the remainder do not serotype, but this may be due to the relatively low overall risk of malignant transformation as well as paucity of services for tissue serotyping compared to general histopathological testing and expenses. In addition, for patients undergoing more than one operation per year, initial histopathology with serotyping would be gold standard but for repeat surgeries with a short interoperative interval, the authors suspect repeat specimen analysis is more sporadic. Recent international consensus statements for JO-RRP recommend obtaining an initial biopsy for histopathological confirmation and considering serotyping, but they do not provide guidance on routine repeat serotyping; repeat pathological evaluation is advised in the context of progressive disease or new suspicious lesions [16]. Moreover, within the current treatment paradigm, HPV serotyping ultimately has minimal impact on definitive management and use of adjuvant therapies for RRP. At present, the literature is ill-defined and clear guidelines do not exist to aid clinicians on the role of repeat biopsies and serotyping.

Alongside multiple airway options come various resection methods, too, such as laser and micro-debrider [17]. CO2 was the first laser to gain widespread popularity in RRP management but since its introduction, a variety of both ablative and photoangiolytic lasers have become more widely available [18]. Our study found that the preferred surgical method varied between JO-RRP and AO-RRP patients (Fig. 2). Microdebrider was the favoured method in JO-RRP and KTP laser for AO-RRP patients. CO2, KTP and blue light lasers together represented the first choice for 41.2% and 46.0% of clinicians for JO-RRP and AO-RRP patients, respectively. The decreased preference for cold steel resection may be due to higher complication rates and poorer voice outcomes compared to newer technologies such as laser [17]. Historically, the most used lasers have been CO2 and KTP [17, 19] and whilst a clearly superior option is yet to be established in the literature, recent systematic reviews have found KTP laser excision better than CO2 when comparing complication and cure rates [20]. Given the varied availability of different lasers and surgical tools across Australia, the results for preferred surgical modality are unlikely to be an accurate representation of surgeon preference alone. It is unclear as to why, however anecdotally paediatric disease is usually higher volume, which could account for the use of microdebrider over laser.

Surgical resection of anterior commissure disease remains an area of uncertainty. Anterior commissure RRP causes significant disease burden, particularly through impacts on voice, however aggressive resection is controversial due to risks of scarring and potential for anterior glottic web formation [21]. Our survey found that most respondents would treat one side only, followed by assessment for vocal improvement before proceeding to a staged contralateral procedure.

More recently, intraoperative adjuvants have been increasingly used in parallel with traditional surgical resection. Several agents have been studied however our survey established that the most popular amongst Australian Otolaryngologists are intralesional bevacizumab and cidofovir. There are a growing number of studies supporting the safety and efficacy of these intralesional adjuvants in both adult and paediatric populations [22, 23]. Vascular endothelial growth factor A is known to play role in the development of RRP with studies showing strong expression on the epithelial layer of respiratory papilloma. Bevacizumab is a monoclonal antibody that inhibits vascular endothelial growth factor A subsequently reducing angiogenesis and microvascular growth [24, 25]. An earlier intralesional adjuvant utilising a markedly different mechanism of action, Cidofovir, is a monophosphate nucleotide analogue that decreases the efficiency of DNA transcription after incorporation into the viral DNA chain. Studies comparing these adjuvants showed that bevacizumab had lower rates of recurrence as well as fewer adverse events [26], perhaps justifying its popularity within this survey with intralesional bevacizumab being twice as favoured as cidofovir.

Alongside intra-operative adjuvants, it is the authors’ opinion that HPV immunisation should be considered in all patients with RRP with recurrent disease after initial surgical debridement. The HPV immunisation was first included in the Australian National immunisation Program in 2007 for cervical carcinoma and since its introduction, national surveillance programs have found a significant decrease in overall incidence of RRP [27, 28]. The efficacy of HPV vaccination has been further supported by recent studies showing a recurrence free interval of more than 3.5 times in RRP patients that were vaccinated compared to those that were unvaccinated in both AO-RRP and JO-RRP [29–31]. Amongst our cohort of Australian ENT consultants, the HPV vaccine was recommended by the majority of clinicians (93.1% in JO-RRP and 81.0% in AO-RRP) and was considered the most common contemporary intervention with the largest impact on RRP management over the course of their careers. The Australian National Immunisation Program currently includes the nonavalent HPV vaccination, ‘Gardasil 9’ which superseded the quadrivalent HPV vaccination, ‘Gardasil’, in 2017. Both vaccinations provide protection against HPV subtypes 6, 11, 16 and 18, covering the subtypes most associated with RRP however Gardasil 9 offers wider protection to additional subtypes more closely associated with conditions other than RRP. Research is scarce comparing outcomes between these two vaccinations in relation to RRP. Despite this, our study found vaccination recommendations differed between AO-RRP and JO-RRP groups. JO-RRP patients were equally recommended the 9-valent and quadrivalent vaccine while AO-RRP patients tended to get recommended the quadrivalent vaccination. Although there are no current guidelines within Australian Laryngological bodies, both the British Laryngological Association and the American Academy of Otolaryngology-Head and Neck Surgery have position statements recommending HPV vaccination of adults to limit RRP. We expect that with time and further research, more robust literature will better define the role of HPV vaccination in the prevention and management of RRP.

### Secondary aim – impact of post-fellowship subspecialty training on RRP assessment and management

Otolaryngology Head and Neck Surgery training programs are variable across Australian states and territories in regard to exposure to patients with RRP and their operative management. Some consultants choose to go onto further training nationally or internationally to gain greater experience at high-volume centres that manage less common pathologies. Laryngology is one of the younger sub-specialties to have developed fellowship opportunities, perhaps only being available in the early 1990s. Many surgeons managing laryngeal pathology would have done so via a paediatric fellowship for JO-RRP, or a head and neck surgery fellowship for AO-RRP. The case mix in these training positions would have been variable, including large amounts of extra-laryngeal diseases. For this reason, a review of the impact of post-fellowship subspecialty training, laryngology or other, was undertaken as a secondary aim.

Limited studies have characterised fellowship trends amongst Australian Otolaryngologists however studies observing US Otolaryngology trainees have found an increased tendency towards fellowship training over time [32]. Most survey respondents had 10 or more years of consultant experience and had undergone further subspecialty training (Table 1; Fig. 1). The most common subspecialty areas in order of frequency were Head and Neck, Laryngology and Paediatric Otorhinolaryngology. On the contrary, those who have been in practice under 10 years were most likely fellowship trained in paediatric ORL or laryngology. Perhaps this trend and predominance of fellowship trained survey respondents is due to a wider availability of sub-specialty fellowships and the shift towards subspecialisation of surgical specialties in general. It also suggests the goal of enhancing patient care, with higher volumes of low-incidence disease being managed by specific centres and surgeons with specific training, such as in the case of RRP. This was also upheld by our data presented in Fig. 3, in which 57.1% laryngology fellowship trained participants would see at least 10 patients with RRP per year, compared to 5.1% of non-laryngology fellowship trained respondents. There is significant evidence across other medical specialties documenting better outcomes for patients being managed by high volume subspecialty units [33, 34].

With reference to the choice of airway and tool for RRP removal, laser was three times more popular than microdebrider in those who had undergone fellowship training in laryngology. Unsurprisingly, for low-volume disease a laser tube was more popular than a microlaryngoscopy tube in this population (35.7% vs. 2.9%). Results were similar in high-volume disease. Given trends toward adjuvant treatment, it also fits that laryngology trained specialists were more likely to use adjuvants (14/14, 100%) compared to non-laryngology fellowship trained surgeons (19/33, 57.6%) who completed this part of the questionnaire. There are many studies demonstrating the superior outcomes with adjuvant therapy [23, 24, 26, 32] and it makes sense that those with greater experience managing RRP would utilise adjuvant therapy if available.

Regardless of disease bulk, most patients’ main functional goals relate to voice use. As such, all participants tended to use stroboscopy and refer for voice therapy perioperatively (Table 4). Practicing with a voice-centred approach, those who underwent laryngology fellowships accordingly tended to use stroboscopy (92.9%), refer for voice therapy (85.7% sometimes, often, or always) and conduct perioperative voice handicap index questionnaires (92.9%). In the non-laryngology fellowship group, these figures were 37.5%, 87.5% and 31.3% respectively.

**Table 4:** Post-operative care of RRP patients with reference to stroboscopy use and voice therapy

Post-operative care	*N* = 43 [percentage of respondents]			
	Never	Sometimes	Often	Always
***Stroboscopy***	18 [41.9]	10 [23.3]	7 [16.3]	8 [18.6]
***Voice therapy***	6 [14.0]	22 [51.2]	11 [25.6]	4 [9.3]

### Comparison to international practices in the management of RRP

RRP management has evolved over the years, with both similarities and differences evident across international practices, when compared with one another and with the Australian cohort.

There is consistency in the current literature that the underlying aetiology is the Human Papillomavirus, JO-RRP is often more aggressive than AO-RRP and types 6 and 11 are the main disease serotypes. As in our Australian survey, international studies have also consequently recommended HPV vaccination [35–38]. Another shared finding across these international studies and our data is the need for multi-modal management in recurrent disease. While surgery remains the cornerstone of treatment, adjuvants such as cidofovir and bevacizumab are common [35, 37]– [38]. One other consistency is the heterogeneity in surgical methods and outcomes, highlighting both the limitations in the current evidence base regarding optimal technique, and the unpredictable nature of the disease as well as the influence of individual surgeon preference on management choices.

Despite these shared themes, important distinctions remain. The primary management of RRP remains surgical removal, but there is a clear trend toward less invasive and more tailored approaches. Murono [35] highlights global variation in surgical technique and emphasizes the need for adjunctive pharmacologic or immunologic therapies given high recurrence rates, suggesting microdebrider is most common in the USA for bulky disease, whereas, in Japan, traditional cold steel remains the preferred approach. A recent international paediatric consensus statement reinforces this heterogeneity, noting that while microdebrider and cold steel techniques were used more frequently than laser modalities across centres, there remains no clear consensus on the optimal surgical approach [16]. An Italian review by Bertino et al. [37] described initial CO₂ laser use under general anaesthesia, progressing to office-based photoangiolytic laser for recurrent disease, along with adjuvant therapy. This is similar in the USA, where in-office laser treatment has been performed for RRP for some time [17]. Although this context was omitted from the survey of Australian Laryngologists, there are access, training and staffing limitations on in-office procedures in Australia and therefore procedures are largely done under general anaesthetic. Similarly, the UK registry database paper does not describe the role of in-office procedures for RRP [38].

## Conclusion

Our findings reflect the current trends of pre-operative, intraoperative and post-operative management of RRP amongst Australian Otolaryngology consultants. Despite being a relatively rare disease, RRP presents a significant disease burden to the global population emphasising the importance of how it is managed. In recent decades, contemporary management options such as new ablative and angiolytic lasers and adjuvant options such as intralesional bevacizumab and the HPV vaccination have changed the way we manage RRP, particularly amongst the laryngology fellowship trained subspecialty population. In Australia, one key area requiring development is the use of office-based procedures for RRP. Whilst our study has highlighted several commonalities in preferences of Australian Otolaryngology consultants, it has also brought to light areas of variability in practice both in Australia and internationally. Although several factors may be contributing to these differences, further research is needed to refine the way we investigate and manage RRP.

## Appendix

List 1

1. Which state do you practice in?
2. How long have you been practicing?
3. Do you practice in a recognised Ear, Nose & Throat Surgery teaching hospital?
4. What is your extent of post FRACS training?
5. How many individual patients with RRP have you seen per year on average over recent years?
6. How many individual patients with RRP do you operate on per year?
7. Do you more commonly operate on Juvenile RRP (< 16 years old) or Adult RRP?
8. Do you recommend the Human Papillomavirus (HPV) vaccine for JORRP patients?
9. Which one?
10. At what age do you recommend it?
11. Do you recommend HPV vaccine for your AORRP patients?
12. If yes, which one?
13. What is your preferred airway for glottic papillomata surgery in low-volume disease?
14. What is your preferred airway for glottic papillomata surgery in high-volume disease?
15. How frequently do you assess the trachea at the time of surgery?
16. How frequently do you assess the main bronchi at the time of surgery?
17. In an individual patient, do you perform a biopsy at each operation?
18. When you do biopsy RRP, do you serotype your specimens?
19. In order, what methods of surgery do you prefer to use in AORRP? (Rank it in order, N/A where necessary)
20. In order, what methods of surgery do you prefer to use in JORRP? (Rank it in order, N/A where necessary)
21. Do you use adjuvant therapy?
22. What is your preferred adjuvant therapy?
23. What is your preferred dose of bevacizumab?
24. Why don’t you use adjuvant therapy?
25. Have you ever referred a patient for systemic bevacizumab?
26. What was your indication for systemic bevacizumab?
27. How do you manage disease at the anterior commissure?
28. What is the most important factor that would persuade you to proceed with surgery?
29. Once macroscopically clear of disease, do you routinely follow-up these patients?
30. How often do you follow up patients clear of disease?
31. When assessing your patients, do you perform stroboscopy for glottic papilloma?
32. How useful do you perceive stroboscopy to be in assessment and management of RRP patients?
33. How often would you refer your RRP patients for post-operative voice therapy?
34. How useful do you think voice therapy is in RRP management? (tick all that apply)
35. Do your patients carry out perioperative patient reported outcome measures for voice (for example, the Voice Handicap Index)?
36. What changes, if any, have you observed over the course of your career with relation to RRP? (tick all that apply)
37. If any, what do you think has had the biggest impact on improving RRP treatment over your career? (tick all that apply)
38. Do you consider optimal management of the RRP population should be undertaken by only those ENT surgeons with: (tick all that apply)
39. Please comment on any other considerations you take into account when deciding whether to operate and how best to manage RRP disease in your patients.
40. Any further comments?
